# Physical exercise elicits UPR^mt^ in the skeletal muscle: The role of c-Jun N-terminal kinase

**DOI:** 10.1016/j.molmet.2023.101816

**Published:** 2023-10-10

**Authors:** Rodrigo Stellzer Gaspar, Carlos Kiyoshi Katashima, Barbara Moreira Crisol, Fernanda Silva Carneiro, Igor Sampaio, Leonardo dos Reis Silveira, Adelino Sanchez Ramos da Silva, Dennys Esper Cintra, José Rodrigo Pauli, Eduardo Rochete Ropelle

**Affiliations:** 1 Laboratory of Molecular Biology of Exercise (LaBMEx), School of Applied Sciences (FCA), University of Campinas (Unicamp), Limeira, Brazil; 2 Laboratory of Cell Signaling, Obesity and Comorbidities Research Center (OCRC), University of Campinas (Unicamp), Campinas, São Paulo, Brazil; 3 Department of Structural and Functional Biology, Biology Institute, University of Campinas (Unicamp), Campinas, Brazil; 4 School of Physical Education and Sport of Ribeirão Preto, University of São Paulo (USP), Ribeirão Preto, São Paulo, Brazil; 5 Laboratory of Nutritional Genomics (Labgen), School of Applied Sciences (FCA), University of Campinas (Unicamp), Limeira, Brazil; 6 Faculty of Medical Sciences, Department of Internal Medicine. University of Campinas (Unicamp), Campinas, São Paulo, Brazil

**Keywords:** Physical exercise, Mitochondria, UPR^mt^, JNK, Skeletal muscle

## Abstract

**Objective:**

The mitochondrial unfolded protein response (UPR^mt^) is an adaptive cellular response to stress to ensure mitochondrial proteostasis and function. Here we explore the capacity of physical exercise to induce UPR^mt^ in the skeletal muscle.

**Methods:**

Therefore, we combined mouse models of exercise (swimming and treadmill running), pharmacological intervention, and bioinformatics analyses.

**Results:**

Firstly, RNA sequencing and Western blotting analysis revealed that an acute aerobic session stimulated several mitostress-related genes and protein content in muscle, including the UPR^mt^ markers. Conversely, using a large panel of isogenic strains of BXD mice, we identified that BXD73a and 73b strains displayed low levels of several UPR^mt^-related genes in the skeletal muscle, and this genotypic feature was accompanied by body weight gain, lower locomotor activity, and aerobic capacity. Finally, we identified that c-Jun N-terminal kinase (JNK) activation was critical in exercise-induced UPR^mt^ in the skeletal muscle since pharmacological JNK pathway inhibition blunted exercise-induced UPR^mt^ markers in mice muscle.

**Conclusion:**

Our findings provide new insights into how exercise triggers mitostress signals toward the oxidative capacity in the skeletal muscle.

## Highlights

• Physical exercise induces mitostress signals in muscle.
• Exercises elicits UPRmt in skeletal muscle.
• JNK coordinates UPRmt in response to exercise.

## 1 Introduction

Mitochondria are the cell powerhouse, which plays a major role in energy production, whole-cell metabolism, apoptosis, whole-body health, and even lifespan [[1], [2], [3]]. Even though it has a bacterial ancestor, discussed within the endosymbiotic hypothesis, the mitochondrial DNA (mtDNA) is responsible for just a few proteins that constitute this organelle (around 13 that form part of the oxidative phosphorylation complexes). At the same time, the majority (over 1400) is derived from nuclear DNA (nDNA) [4,5]. Thus, any adaptations that ensure optimal mitochondrial function require strict coordination between mitochondrial and nuclear genomes, involving integrated signaling toward transcription, translation, translocation, and import processes [6,7]. This precise, integrative, dynamic process – also known as mitonuclear communication – and its proper functioning are essential to sustain cellular homeostasis and spark stress-responsive signals and any needed adaptations [8,9].

Many sources of stress and proteotoxic damage frequently threaten mitochondrial proteostasis, requiring a sophisticated system to ensure protein quality control [6]. The hunk of unfolded proteins (most mitochondrial proteins must be imported from the cytoplasm) can be an important stress catalyst. Most complexes from the electron transport chain (ETC) contain subunits originating from nDNA and mtDNA [8,10]. Consequently, they have precise stoichiometric ratios that assure ideal mitochondrial function. Certain circumstances might trigger stoichiometric imbalance between mtDNA-encoded proteins (such as MTCO1) and nDNA-encoded subunits (such as ATP5A or SDHA), affecting mitochondrial function, in a response called mitonuclear imbalance [11]. To reinforce such a flawless state, chaperones (such as Hsp60) might act by folding the freshly imported proteins or even refolding glitched proteins, while proteases, including ClpP, Lonp1, and Yme1L1 assure the eradication of proteins irreversibly impaired [12,13]. Under stress, mitochondria communicate with the nucleus, triggering a transcriptional response that accounts for mitonuclear imbalance [11,[14], [15], [16]] performance and longevity in different experimental models.

Physical exercise is a well-known potent stimulus for mitochondrial biogenesis and function [[17], [18], [19]], due to its stressor effects on the whole body, by increasing oxygen and energy demands, thus leading to multiple adaptations related to health and performance [20]. Numerous stimuli of muscle contraction, which create transient increases in mRNA and, subsequently, protein levels [21], are considered essential for a metabolic shift due to a training regimen. Importantly, it has been reported that physical exercise increases the intracellular levels of stimulates Nicotinamide Adenine Dinucleotide (NAD) [22,23] and NAD-dependent deacetylase sirtuin-1 (SIRT1) and Peroxisome proliferator-activated receptor gamma coactivator 1-alpha (PGC-1α) activity, improving mitochondrial function [16,24]. However, the effects of physical exercise on UPR^mt^ markers are poorly understood.

In this context, we sought to determine the impact of acute and chronic physical exercise on UPR^mt^ in the skeletal muscle of healthy young mice. In addition, we explored the mechanism by which exercise modulates UPR^mt^.

## 2 Results

### 2.1 Physical exercise modulates mitostress-related genes in muscle

**Figure 1:**
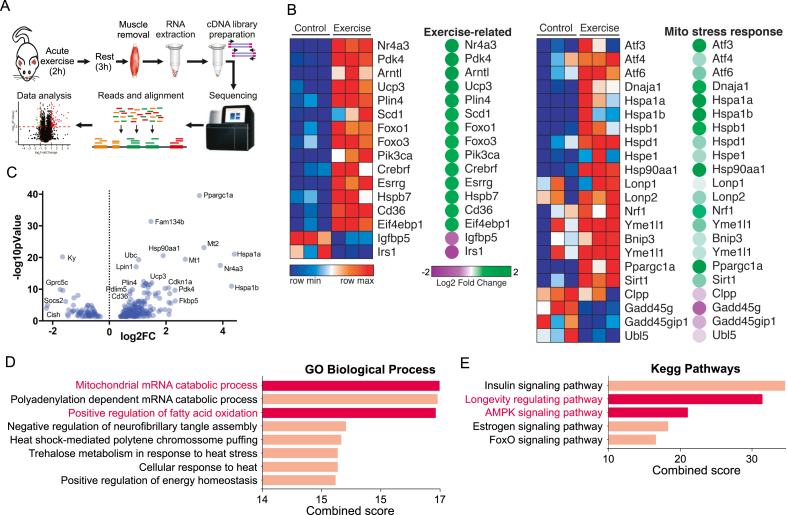
Transcriptome from the skeletal muscle of exercised male mice. (A) Experimental design. (B) Heatmap using transcripts from control and exercised mice. Gastrocnemius samples were obtained 3 h after the acute swimming exercise (n = 3). (C) Volcano plot. (D) GO biological process. (E) KEGG pathways analyses.

To better understand the effects of physical exercise on mitochondrial stress-related genes, male C57BL/6j mice performed a single session of prolonged aerobic exercise [25]. The gastrocnemius muscle was removed 3 h later for RNA sequencing assay (Figure 1A). Transcriptome analysis confirmed that acute swimming increased several exercise-related genes (Figure 1B). Importantly, acute aerobic exercise stimulates a consistent list of mitostress-related genes, including the UPR^mt^ markers (*Hspd1*, *Lonp1,* and *Yme1l1*) (Figure 1B). The volcano plot revealed that acute exercise markedly increased the expression of genes related to mitostress, in particular, chaperones (*Hsp90aa1*, *Hspa1a,* and *Hspa1b*), metalloproteinases (*Mt1* and *Mt2*) and other critical genes related to mitochondrial function (*Ppargc1a*, *Ucp3*, *Pdk4,* and others) (Figure 1C). The Gene Ontology (GO) analysis confirmed that exercise increased a group of genes related to mitochondrial stress and function in mouse skeletal muscle (Figure 1D). In addition, the KEGG pathway database also indicated that a group of genes induced by acute exercise involve mitochondrion-related pathways, including longevity-related pathways and AMPK signaling (Figure 1E). This initial screening confirmed that physical exercise acutely triggers stress-related signals toward mitochondria.

### 2.2 Acute exercise elicits UPR^mt^ in skeletal muscle

**Figure 2:**
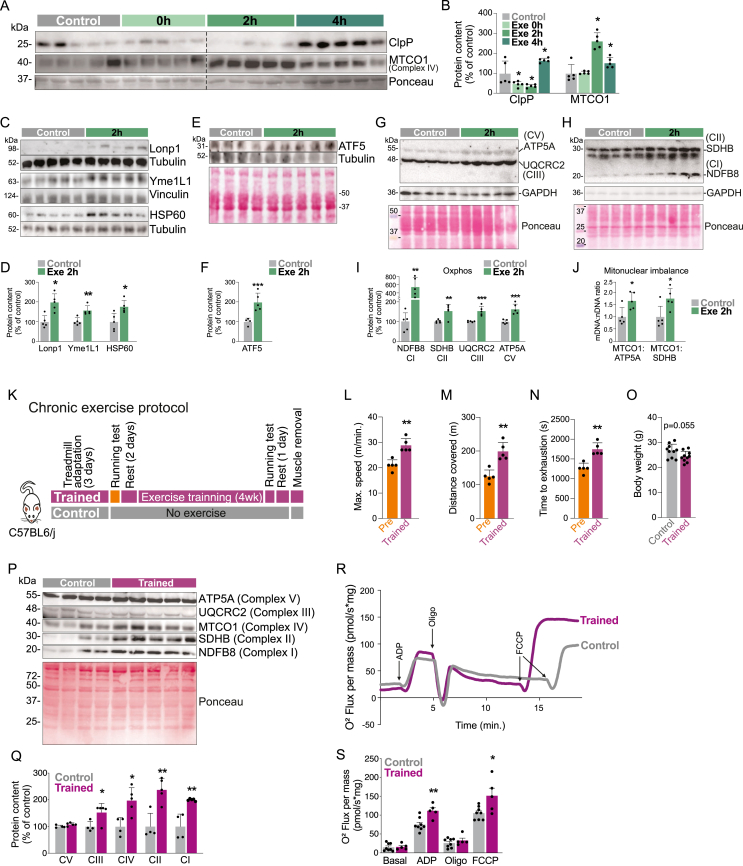
Acute treadmill running elicits UPR^mt^ in the skeletal muscle of male mice. Western blot and respective quantification for (A and B) ClpP and MTCO1 (n = 5, ∗p < 0.05 vs. control). (C and D) Lonp1, Yme1L1 and HSP60 (n = 5, ∗p < 0.05 and ∗∗p < 0.01 vs. control). (E and F) ATF5 protein content (n = 4–5, ∗∗∗p < 0.001 vs. control). (G–I) OXPHOS components (n = 5, ∗∗p < 0.01 and ∗∗∗p < 0.001 vs. control). (J) Mitonuclear imbalance was obtained by MTCO1/ATP5A and MTCO1/SDHB stoichiometric ratio. The Western blots from Figures A, G, and H were used for mitonuclear imbalance calculation (n = 5, ∗p < 0.05 vs. control). (K) Experimental design for chronic exercise. (L) Maximal speed, (M) distance covered, and (N) time to exhaustion during the running test (n = 5, ∗∗p < 0.01 vs. control). (O) Body weight after the running training (n = 10). (P and Q) Western blot and respective quantification for OXPHOS (n = 4–5, ∗p < 0.05 and ∗∗p < 0.01 vs. control). Mitochondrial respiration: (R) representative curve of oxygen consumption and (S) mitochondrial respiration under basal condition and after ADP, oligomycin, and after FCCP administration (n = 5–8).

Beyond the gene expression, we next sought to confirm mitostress signals in the skeletal muscle, monitoring the protein content of UPR^mt^ markers using acute treadmill running protocol. The time-course study combined with Western blot analysis revealed that acute treadmill exercise rapidly stimulated the mtDNA-encoded protein, MTCO1, a critical component of the complex IV of the electron transport chain, 2 h after the exercise protocol (Figure 2A,B). The protein content of mitochondrial protease, ClpP (a UPR^mt^ marker), was increased 4 h after the exercise (Figure 2A,B). The treadmill running also increased the protein content of other UPR^mt^ markers in the skeletal muscle, including the mitochondrial proteases, Lonp1 and Yme1L1, and chaperone HSP60 (Figure 2C,D). Furthermore, the stoichiometric ratio between the ETC component originating from mtDNA (MTCO1) by ETC components originating from nDNA (ATP5A and SDHB) revealed that exercise also induced the mitonuclear imbalance (Figure 2J). These data were accompanied by high levels of ATF5 protein content in the skeletal muscle (Figure 2E,F), confirming the presence of the UPR^mt^ mechanism in the skeletal muscle. Acute exercise also increased several ETC components, such as ATP5A (CV), MTCO1 (CIV), UQCRC2 (CIII), SDHB (CII), and NDUFB8 (CI) in the skeletal muscle (Figure 2A, G, and H). These data demonstrate that acutely, swimming and treadmill exercise protocols elicit the UPR^mt^ in the skeletal muscle at the genetic and protein level.

Next, to evaluate the physiological adaptations in response to the chronic UPR^mt^ activation, mice were submitted to 4-week aerobic treadmill running (Figure 2K). Trained mice presented improvement in their aerobic capacity (Figure 2L–N). No change was observed in the total body weight (p = 0.055) (Figure 2O). Gastrocnemius submitted to chronic exercise had increased protein contents of OXPHOS components, MTCO1 (CIV), UQCRC2 (CIII), SDHB (CII), and NDUFB8 (CI) (Figure 2P,Q). Importantly, skeletal muscle tissue from trained mice also presented increased mitochondrial respiration when exposed to ADP (state 3 respiration) and when evaluated maximal respiratory capacity state U, using chemical uncoupler FCCP (Figure 2R,S). These findings suggest that long-term UPR^mt^ activation can be associated with mitochondrial adaptatiom in the muscle fibers.

### 2.3 Low levels of UPR^mt^-related genes in skeletal muscle affect exercise-related phenotypes in BXD strains

**Figure 3:**
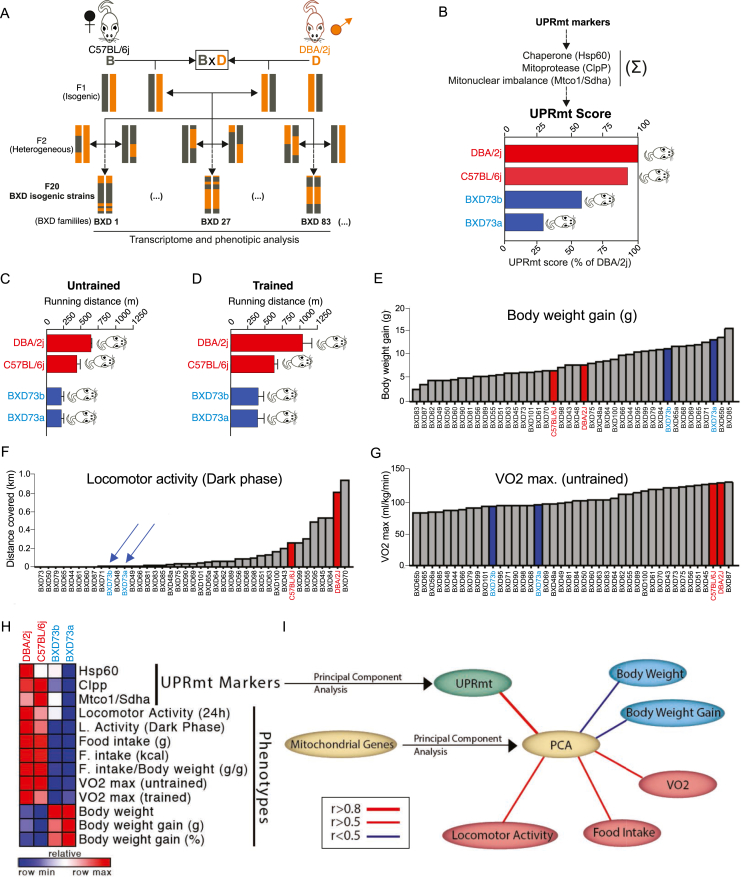
Bioinformatics analysis from BXD mouse reference population. (A) Inbred model for BXD strains generation. (B) Identify strains with high and low scores for UPR^mt^ gene expression in the skeletal muscle of male mice. (C and D) Running distance in untrained and trained mice, respectively. (E) Body weight gain, (F) locomotor activity during the dark phase, and (G) VO2max in 42 BXD strains of mice. (H) Heat map (I) Multiple correlation graph using the BXD database, including transcriptome data from skeletal muscle and several phenotypes. Blue lines represent negative and red lines represent positive correlations. Pearson's correlations were applied.

The BXD mouse genetic reference is considered the largest and best-categorized family of isogenic strains and has been widely used in the genetic analysis of numerous complex physiologic phenotypes. The BXD recombinant inbred mice are descended from C57BL/6j and DBA/2J progenitor strains. Heterogeneous F2 generations were crossed (for >20 generations), generating the BXD isogenic strains (Figure 3A).

Initially, the gene expression pattern in the skeletal muscle of 42 BXD strains was evaluated. Once UPR^mt^ is a mechanism dependent on a multiplicity of proteins, including proteases and chaperones, we developed a “*UPR*^*mt*^ *Score*” using the gene expression of UPR^mt^ markers, including *Hspd1* (HSP60), *Clpp*, gene expression, and the presence of the mitonuclear imbalance, defined by *Mtco1/Sdha* ratio (Figure 3B). This screening revealed that, while progenitor strains (C57BL/6j and DBA/2J) presented high UPR^mt^ scores, BXD73a and BXD73b families displayed very low UPR^mt^ scores in the skeletal muscle (Figure 3B). Interestingly, BXD73a and 73b mice showed low aerobic performance during the running test, before and after training (Figure 3C,D). These strains also presented higher weight gain, lower locomotor activity, and VO_2_max than C57BL/6j and DBA/2J mice (Figure 3E–G). The heatmap analysis confirmed that low levels of UPR^mt^-related genes in skeletal muscle affect exercise-related phenotypes (Figure 3H).

Using the transcripts from 42 mouse strains, the Principal Component Analysis (PCA) demonstrated that UPR^mt^-related genes in skeletal muscle are strongly and positively correlated with several mitochondrial-related genes in the skeletal muscle, driving better exercise-related phenotypes, including locomotor activity and VO_2_max (Figure 3I). Thus, the BXD mouse genetic reference populations confirmed that UPR^mt^-related genes in skeletal muscle are closely linked to exercise-related phenotypes.

### 2.4 Acute exercise activates JNK signaling in skeletal muscle

We next sought to determine if the c-Jun N-terminal kinase (JNK) pathway was involved in exercise-induced UPR^mt^ in the skeletal muscle. JNK pathway was considered a potential target once this kinase can be activated by different internal or external stressful stimuli [26,27], including muscle contraction [28,29]. In addition, it has been proposed that JNK coordinates mitochondrial stress in mammalian cells [9].

**Figure 4:**
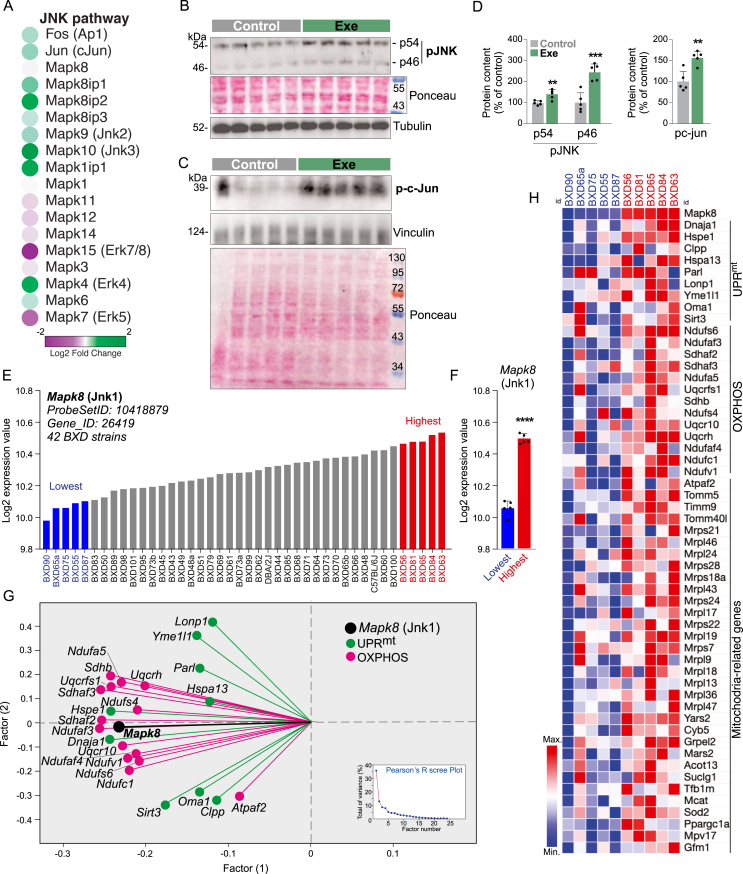
Evaluation of JNK signaling in the skeletal muscle of male mice. (A) Transcripts from the skeletal muscle of mice submitted to acute swimming exercise (n = 3). (B–D) Western blot and respective quantification for JNK signaling in the skeletal muscle of mice submitted to acute treadmill running (n = 5, ∗∗p < 0.01 and ∗∗∗p < 0.001 and vs. control). (E) *Mapk8* gene expression in the skeletal muscle of 42 BXD strains. (F) *Mapk8* gene expression in the skeletal muscle (n = 5, ∗∗∗∗p < 0.0001 and vs. Lowest). (G) Two-factor analysis using the whole database from the skeletal muscle of BXD mice. Pearson's R scree plot was applied. (H) Heatmap using transcripts from muscle of BXD mice.

Initially, our transcriptome data revealed that acute swimming exercise increased the gene expression of *Mapk9* (Jnk2), *Mapk10* (Jnk3), and the gene expression of transcription factors downstream of JNK signaling, such as *Fos* (Ap1) and *Jun* (cJun) in the skeletal muscle C57BL/6j male mice (Figure 4A). Beyond the gene expression, we also found that acute treadmill running stimulated JNK (p54 and p46) threonine phosphorylation and cJun (Serine 63) phosphorylation in muscle fibers (Figure 4B–D). We observed that the same acute exercise also stimulated UPR^mt^ markers in the muscle of female mice (Supplementary Figure 1A, B).

After that, 42 BXD strains were sorted regarding *Mapk8* gene expression in the skeletal muscle. Five strains with the lowest (blue) and 5 strains with the highest (red) *Mapk8* gene expression were identified (Figure 4E,F) and analyzed. The 5 strains with low *Mapk8* (Jnk1) gene expression (BXD90, 65a, 75, 55, and 87) presented low levels of UPR^mt^, OXPHOS, and other mitochondrial-related genes in the skeletal muscle. Conversely, the 5 strains with high levels of *Mapk8* (Jnk1) gene expression (BXD56, 81, 65, 84, and 63) displayed an opposite pattern of gene expression in the skeletal muscle (Figure 4H). Two-factor analysis using the whole BXD mouse cohort confirms that *Mapk8* (Jnk1) is strongly associated with several UPR^mt^ markers and OXPHOS-related genes in muscle (Figure 4G). Thus, bioinformatics and molecular analysis *in vivo* strongly suggest that JNK signaling can be involved in UPR^mt^ activation in the skeletal muscle.

**Figure 5:**
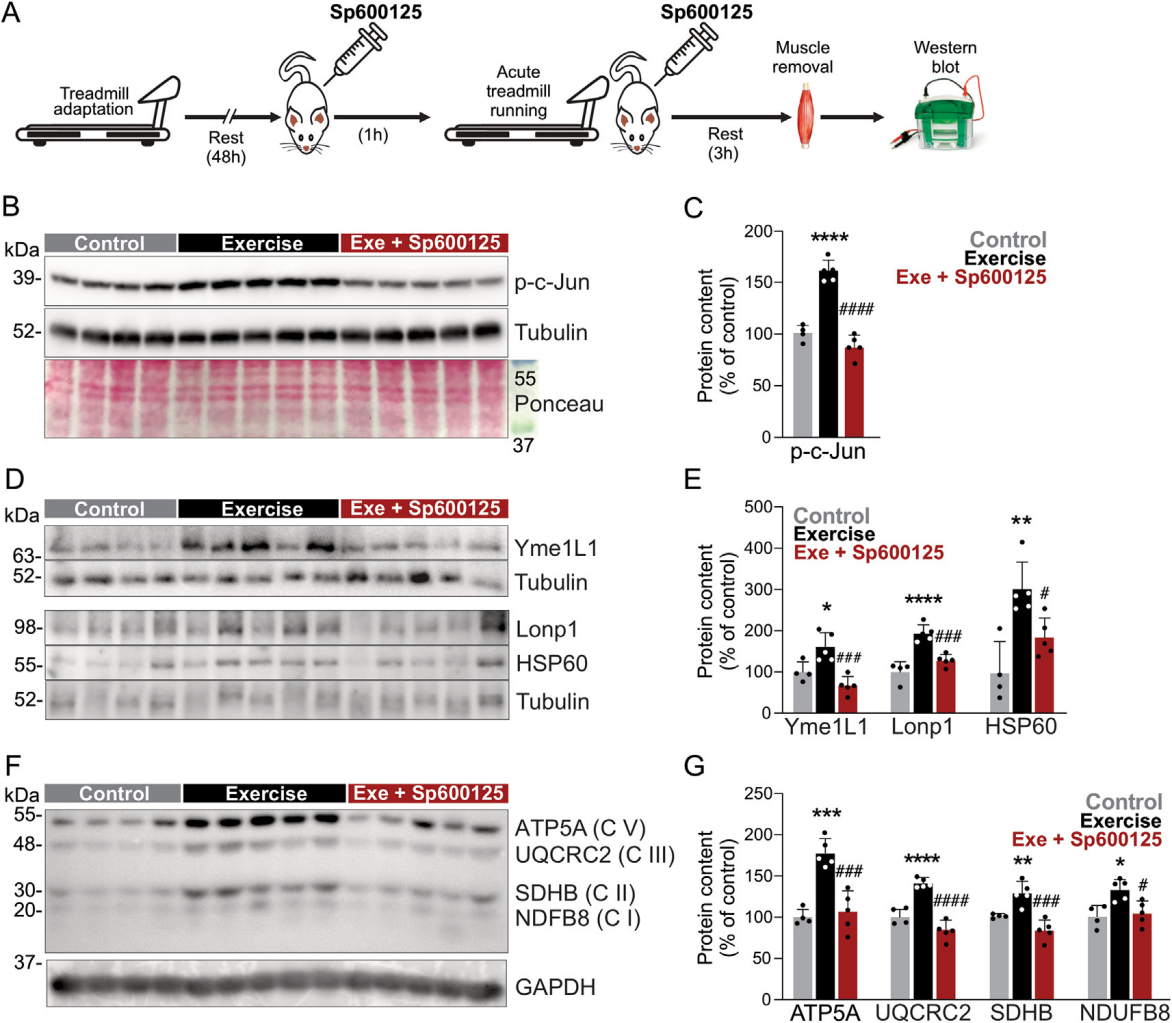
Effects of pharmacological JNK inhibition in the muscle of exercised male mice. (A) Experimental design. (B–G) Western blot and respective quantification for p-c-Jun, Yme1L1, Lonp1, HSP60, and OXPHOS components in the skeletal muscle (n = 4–5, ∗p < 0.05, ∗∗p < 0.01, ∗∗∗p < 0.001 and ∗∗∗∗p < 0.0001 vs. control or ^#^p < 0.05, ^###^p < 0.001 and ^####^p < 0.0001 vs. exercise).

To confirm if the JNK pathway is involved in exercise-induced UPR^mt^ activation, a pharmacological study *in vivo* was conducted. The pharmacological JNK inhibitor (SP600125) was intraperitoneally injected in mice 1h before and immediately after the acute treadmill running, and the skeletal muscle samples were obtained 3 h later (Figure 5A). The acute pharmacological JNK signaling inhibition did not affect the exercise performance once both groups could complete the acute exercise protocol similarly. The Western blot analysis confirmed that SP600125 administration effectively inhibits exercise-induced JNK pathway activation in the skeletal muscle (Figure 5B,C). Interestingly, the pharmacological JNK inhibition attenuated the UPR^mt^ activation in the muscle of exercised mice (Figure 5D,E). Finally, while acute exercise increased the content of several OXPHOS-related proteins in the skeletal muscle, SP600125 abolished this effect (Figure 5F,G), confirming the critical role of JNK driving the mitostress signals in muscle fibers in response to exercise.

## 3 Discussion

Physical exercise has been associated with various adaptations, which are mediated by increased muscle function, mitochondrial biogenesis, and oxidative capacity [21]. Nevertheless, the influence of UPR^mt^ on these responses remains unclear. In the current study, we evaluated the role of exercise on UPR^mt^ induction in the skeletal muscle of male and female mice. In addition, we investigated the involvement of JNK in exercise-induced UPR^mt^ activation.

Increased SIRT1 activity has been reported to stimulate mitochondrial biogenesis [15,30], increasing the activity of the enzymes involved with the electron transport chain and beta-oxidation, allowing the skeletal muscle to have increased ATP production and exercise capacity [31]. Increased SIRT1 activity has also been proven to disturb the stoichiometric balance between mtDNA and nDNA-encoded OXPHOS subunits of the mitochondrial electron transport chain, known as mitonuclear imbalance [11], which triggers the UPR^mt^. This well-conserved stress response ensures mitochondrial proteostasis and function, being associated with longevity in experimental models [11,32]. Analysis of murine reference populations [33] revealed a positive correlation between UPR^mt^ and longevity, a conserved mechanism in mammals that depended on the mitonuclear imbalance between mtDNA and nDNA-encoded OXPHOS proteins [11]. It was demonstrated to trigger the UPR^mt^, inducing chaperones and proteases to ensure proteostasis and ending with an extended lifespan. Our transcriptome analysis provided consistent data confirming that acute exercise led to the mitonuclear imbalance, stimulating several genes related to mitochondrial stress and longevity-regulating pathways in the skeletal muscle.

In a contractile activity model (which provides more local adaptations when compared to an exercise protocol that systemically affects homeostasis and energy consumption), low-frequency-stimulated skeletal muscle presented increased mitochondrial biogenesis, UPR activation, and mitochondrial chaperones and proteases [34,35], thus reinforcing that the adaptations to exercise and metabolic shift involve mitonuclear imbalance, UPR^mt^, and influence increased metabolic health and fitness. The UPR^mt^ activation in the skeletal muscle was also found in different muscle contraction models [34,[36], [37], [38], [39]]. In the present study, chronic aerobic exercise protocol suggested that physiological and transitory UPR^mt^ stimulation could be associated with the positive adaptation of the mitochondria in muscle. Conversely, two families of BXD mice (73a and 73b) with very low gene expression of different components of UPR^mt^ in the skeletal muscle presented body weight gain and low aerobic capacity along their lives.

Consistent with this notion, chronic UPR^mt^ activation plays a key function in shifting aged stem cells towards optimal functionality, besides preventing its senescence in a mouse aging model [1]. We have recently demonstrated that four weeks of moderate or high-intensity training led to mitonuclear imbalance and UPR^mt^ activation in the skeletal muscle of aged mice [40,41]. Similar data were found in trained young mice in the current study. In addition, MDX mice (an animal model of Duchenne's muscular dystrophy) treated with nicotinamide riboside (NAD^+^ precursor and UPR^mt^ activator) improved muscle function and metabolism, suggesting an important role of NAD^+^ and UPR^mt^ in inducing mitochondrial function pathways [42]. Collectively, these findings strongly suggest that long term of UPR^mt^ activation is tightly associated with mitochondrial adaptation in muscle cells.

The mechanism by which exercise initiates the intracellular signals toward the UPR^mt^ in the skeletal muscle remains only partially known. It has been proposed that mitochondrial reactive oxygen species (ROS) promote the decisive signal for UPR^mt^ activation in the skeletal muscle [43]. In addition, Slavin and colleagues demonstrated that the Activating Transcription Factor 5 (ATF5) plays a critical role in controlling UPR^mt^ markers in the skeletal muscle of exercised mice. Interestingly, acute exercise triggered the accumulation of ATF5 in the mitochondrial fraction [44]. In our findings we observed that acutely, exercise increased JNK phosphorylation and ATF5 protein content in the skeletal muscle. It is plausible that mechanical and/or metabolic stress triggered during exercise could be associated with activating transcription factors related to UPR^mt^ genes in muscle fibers.

In this scenario, we determined the role of the key cellular stress sensor, JNK [27], in the muscle of exercise mice. Accumulating studies have demonstrated that physical exercise and muscle contraction stimulate JNK phosphorylation [28,29]; however, the role of JNK activation in triggering UPR^mt^ in response to exercise is unknown. In the current study, the combination of bioinformatics analysis and experimental approaches revealed that UPR^mt^ activation in the skeletal muscle occurred, at least in part, in a JNK-dependent fashion. The pharmacological JNK inhibitor blunted acute exercise-induced Lonp1, Yme1L1, and HSP60 protein content in the skeletal muscle. In line with our findings, Rath and colleagues reported high levels of JNK phosphorylation and UPR^mt^ activation in Cos-7 cells under proteotoxic stress [45]. It is important to mention that although the BXD strains with low *Mapk8* (Jnk1) gene expression in the skeletal muscle displayed low exercise capacity, the acute JNK inhibition did not affect the exercise performance in C57BL/6j mice, suggesting that acutely JNK and UPR^mt^ activation are not involved in muscle skeletal contraction capacity, but probably in the skeletal muscle adaptation.

Beyond the UPR^mt^, the Integrated Stress Response (ISR) is associated with improving mitochondrial function [46]. UPR^mt^ and ISR are strongly linked, functioning together to maintain mitochondrial function [47]. ISR promotes the reduction of global translation through the phosphorylation of serine 51 residues in the α-subunit of eIF-2 [48]. Curiously, ATF4, ATF5, and CHOP transcription factors are selectively translated during the ISR [46]. Although eIF2-α phosphorylation was not monitored in our study, we found high levels of *Atf3, 4,* and *6* gene expression, as well as ATF5 protein content, in the skeletal muscle after the acute exercise, suggesting the ISR activation. Thus, we cannot exclude the participation of the ISR for mitochondrial adaptation in response to exercise. Finally, our findings link JNK activation with the mitostress in response to exercise, providing new insights into how physical exercise stimulates mitochondrial adaptation in skeletal muscle.

## 4 Methods

### 4.1 Animals

Male and female C57BL/6j mice were obtained from the University of Campinas Central Breeding Center. They were kept in individual cages with controlled temperatures (22–24 °C) and light and dark cycles (12 h). They also had *ad libitum* access to standard rodent chow and water. All studies were subjected to the University of Campinas Animal Ethics Use Committee (number 3809-1).

### 4.2 Acute swimming protocol

Five animals per turn were acclimated to the water for three consecutive days, 10 min a day, in plastic containers of 40 cm in length, 30 cm in width, and 45 cm in depth. The water temperature was maintained at approximately 33 °C during the entire protocol. Afterward, part of the acclimated animals was subjected to a single exercise. The swimming protocol was composed of four sessions of 30 min with 5-min intervals in between, for a total of 2 h, as previously described [25]. To avoid the influence of the stress- and temperature-related water, the animals from the control group performed the acclimation protocol similarly to the animals from the exercise group. In addition, during the exercise protocol, the animals from the control group remained in the tank containing ∼1.5 cm of water. Gastrocnemius muscle samples were obtained 3 h after the exercise protocol.

### 4.3 RNA sequencing

The TruSeq Stranded mRNA Library Prep Kit (Illumina) was used to prepare the libraries. RNA sequencing was performed using HiSeq. 2500 (Illumina) at the Life Sciences Core Facility (State University of Campinas), resulting in around 20 million reads per sample. These results were analyzed for quality, filtered, and trimmed using FASTQC (available at: https://www.bioinformatics.babraham.ac.uk/projects/fastqc/) and Trimmomatic [49]. The Bowtie2 software was used for reads alignment. The RSEM software was used to identify differentially expressed genes (DEGs). Gene Ontology and KEGG analyses were performed using DAVID 6.8 Beta. Heatmaps were created using the GENE-E software (https://software.broadinstitute.org/GENE-E/).

### 4.4 Acute treadmill running protocol

Before the acute running exercise, C57BL/6j mice underwent an adaptation protocol for 3 consecutive days. In the acute physical exercise protocol, mice performed a single exercise session for 60 min at 60 % peak workload. Gastrocnemius muscle samples were obtained 0, 2, and 4 h later.

### 4.5 Physical training protocol

Before the training period, C57BL/6j mice underwent an adaptation protocol for 3 consecutive days to minimize the physical exercise stress. An incremental running test on a motor treadmill determined the maximal lactate steady state. The exercise intensity started at 6 m/min, with 3 m/min increases every 3 min at 0 % grade until exhaustion was reached. This evaluation provided the distance covered and the peak workload, which was later used to determine the animal's 60 % peak workload.

Before the start of the exercise protocol, 8-week-old mice were randomly assigned into sedentary (n = 10) and trained (n = 10) groups. The exercise training protocol was adapted from Ferreira et al. (2007) [50] and consisted of 4 weeks of running on a motor treadmill (ESD model 01; FUNBEC, São Paulo, Brazil), 5 days/week, for 60 min at 60 % peak workload, which corresponds to the maximal lactate steady state workload. Another incremental running test was performed after the exercise training protocol to evaluate the effect of exercise training on running performance and the total distance covered. One day after the last exercise session, mice were anesthetized and had a muscle biopsy taken for Western blotting analysis.

### 4.6 In vivo pharmacological JNK inhibition

One hour before and immediately after the acute physical exercise protocol, mice were injected intraperitoneally with either saline (control) or SP600125 (JNK inhibitor, 15 mg/kg of body weight) as previously published [51].

### 4.7 Oxygen consumption protocol

A sample of the skeletal muscle soleus, obtained from C57BL/6j mice biopsy, was immediately placed in an ice-cold biopsy preservation medium (BIOPS; OROBOROS Instruments, Innsbruck, Austria). The muscle fibers were permeabilized with saponin and transferred into an ice-cold mitochondrial respiration buffer (MiR05; OROBOROS Instruments). Subsequently, the muscle fibers were transferred to the Oxygraph (OROBOROS Instruments) to perform high-resolution respirometry corrected for wet weight.

Mitochondrial function *ex vivo* was determined by polarographically measuring oxygen consumption using a two-chamber Oxygraph. Oxygen consumption/flux reflects the first derivative of the oxygen concentration (nmol/mL) in time in the respiration chambers, which is oxygen flux (pmol/s/mg) and is corrected by the muscle tissue wet weight (2–5 mg) in the chamber. The high-resolution respirometry was adapted from a previously published protocol. To evaluate the mitochondrial oxidative capacity, 4.0 mmol/L malate was added to obtain state 2 respiration, followed by 8.0 mmol/L glutamate addition as a substrate for complex I. Additionally, an excess of 1.6 mmol/L ADP was added to evaluate state 3 respiration, reflecting the substrate oxidation coupled to energy production. After that, 8.0 mmol/L succinate was added to obtain state 3 respiration of complex I and II. Finally, 1.6 μg/mL oligomycin was added to evaluate state 4 respiration, and titrations (in steps of 0.5 μL of 1.0 mmol/L) of the chemical uncoupler fluoro-carbonyl cyanide phenylhydrazone (FCCP) were added to evaluate maximal respiratory capacity state U.

### 4.8 Western blot

Muscle samples were applied in polyacrylamide gel for electrophoresis (SDS-PAGE) separation and transferred to 0.45 μm nitrocellulose membranes. Next, the membranes were blocked for 3 min, and a Ponceau Red (Sigma–Aldrich, Saint Louis, MO, USA) staining was performed. After that, the membranes were blocked with 5 % of non-fat dry milk at room temperature for 1 h and finally incubated with primary antibodies. Specific bands were labeled by chemiluminescence reaction, and visualization was performed by using GeneSys Software®. The Ponceau and protein band images were stored and quantified using the UN-SCAN-IT® software. Tubulin or GAPDH normalized the Western blotting results. In some cases, the Ponceau staining was also used as a loading control.

Anti-OXPHOS (Abcam Plc, Cambridge, United Kingdom; Cat# ab110413, RRID: AB_2629281), anti-HSP60 (Santa Cruz Biotechnology, CA, USA; Cat# sc-13115, RRID: AB_627758), anti-Phospho-cJun (Santa Cruz Biotechnology, CA, USA; Cat# sc-822, anti-Phospho-JNK (Santa Cruz Biotechnology, CA, USA; Cat# sc-6354, anti-ClpP (Santa Cruz Biotechnology, CA, USA; Cat# sc-271284, anti-YME1L1 (Proteintech, Rosemont, Illinois, USA; Cat# 11510-1-AP, RRID: AB_2217459), anti-α-tubulin (Cell Signaling Technology; Cat# 2144, RRID: AB_2210548), anti-Vinculin (Cell Signaling Technology; Cat# 4650), anti-GAPDH (Cell Signaling Technology; Cat# 2118, RRID: AB_561053), anti-LONP1 (Bioss Antibodies, Boston, Massachusetts, USA; Cat# bs-4245R, RRID: AB_11051909) and anti-ATF5 (ABclonal, Cat# A18155). All antibodies were used at dilution 1:1000. Ponceau was from Sigma–Aldrich (Saint Louis, MO, USA). The respective loading control normalized the Western blotting results, and the final values were given in the percentage of the respective control group.

### 4.9 Bioinformatics analysis

Correlation analyses were performed using a database obtained from the skeletal muscle of the BXD inbred family (EPFL/LISP BXD CD Muscle Affy Mouse Gene 1.0 ST (Dec11) RMA; BXD Published Phenotypes). These data sets are accessible on Genenetwork (http://www.genenetwork.org). Pearson's and Spearman's correlation graphs were built using GraphPad Prism, and the heat map graph was obtained using the Gene-E software. The correlation between skeletal muscle UPR^mt^ markers (*Mtco1, Hspd1, Clpp,* and *Sdha*), gene expression, and different BXD phenotypes was performed using distinct datasets.

The UPR^mt^ score was obtained by summing the gene expression of *Clpp, Hspd1*, and the mitonuclear imbalance index resulting from the gene expression ratio of *Mtco1:Sdha* in the skeletal muscle of BXD mice.

### 4.10 Statistical analysis

All the results were expressed as mean ± SD. Immunoblotting results were presented as direct comparisons of the autoradiographic bands and respective quantification. Data were analyzed by Student t-test or one-way ANOVA, as appropriate, with post-hoc Bonferroni tests for multiple unpairwise comparisons of means. The level of significance adopted was *p* < 0.05. GraphPad Prism 6.0 software was used for the analysis. The number of animals used in each experiment was described in the figure legend. For the bioinformatics analysis, Pearson's correlation was used.

## Author contributions

R.S.G. and E.R.R. planned most experiments and wrote the paper. R.S.G. and E.R.R. performed the bioinformatics analysis. R.S.G. and B.M.C. performed the exercise protocol. R.S.G. performed the Western blotting analysis for the chronic exercise protocol and the time-course study. C.K.K. Performed the Western blotting analysis for acute exercise protocols and SP600125 experiment. F.S.C. performed the female experiments. I.S. and L.R.S. contributed to the oxygen consumption determination. A.S.R.S., D.E.C., and J.R.P. contributed to the discussion, laboratory support, and manuscript review.

## Declaration of competing interest

The authors declare that they have no known competing financial interests or personal relationships that could have appeared to influence the work reported in this paper.

## Data Availability

Data will be made available on request.
