# Supplementary material for: Physical exercise elicits UPRmt in the skeletal muscle: The role of c-Jun N-terminal kinase
Source: Mol Metab. 2023 Oct 10;78:101816. doi: 10.1016/j.molmet.2023.101816 (PMC10590869; doi:10.1016/j.molmet.2023.101816)
Supplement: Multimedia component 1 [file mmc1.pdf]

Supplementary figure 1

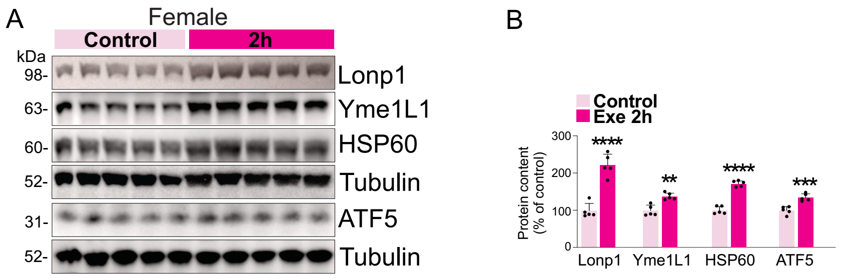

Figure S1. Acute treadmill running elicits UPRmt in the skeletal muscle of female mice. Western blot and respective quantification for (A and B) Lonp1, Yme1L1, HSP60 and ATF5 (n=5, **p<0.01, ***p<0.001vs. control and ****p<0.0001vs. control).
